# Transverse colon volvulus presenting as bowel obstruction, atelectasis, and displacement of the right lobe of the liver into the left upper abdominal quadrant: a case report

**DOI:** 10.1186/s13256-023-03840-1

**Published:** 2023-04-09

**Authors:** Mubaraka Kayiira, Eria Muwanguzi, Derrick Kasozi, Peter Waitt, Rogers Ayebare, Edwin Musinguzi, Innocent Orimunsi, Paul Okeny, Peter Mbide, Timothy Arthur Serumaga, Nicholas Tamale

**Affiliations:** 1 Infectious Diseases Institute, Makerere University College of Health Sciences, Kampala, Uganda; 2 Department of Surgery, Makerere University College of Health Sciences, Kampala, Uganda; 3 Department of Surgery, Fort Portal Regional Referral Hospital, Fort Portal, Uganda

**Keywords:** Transverse colon volvulus, Bowel obstruction, Right lung collapse, Case report, Displacement of the right lobe of the liver, Point of care ultrasonography

## Abstract

**Background:**

Transverse colon volvulus is an uncommon cause of intestinal obstruction. It is a surgical emergency that can lead to bowel infarction, peritonitis, and death.

**Case presentation:**

We report a case of transverse colon volvulus in a 35-year-old Congolese immigrant man who had a rare presentation with features of intestinal obstruction associated with right lung collapse and left mediastinal shift.

**Conclusion:**

This case is unusual because it presented with respiratory features that mimicked a pneumothorax in addition to features of intestinal obstruction. The use of point-of-care lung ultrasound was helpful in ruling out a pneumothorax, and this could help avoid situations such as unintentional chest drain insertions by other professionals who may encounter a similar case. Because transverse colon volvulus is rare, a high level of suspicion and awareness is required to make an accurate diagnosis.

**Supplementary Information:**

The online version contains supplementary material available at 10.1186/s13256-023-03840-1.

## Background

A volvulus is a twisting or axial rotation of a portion of the bowel around its mesentery. When complete it may form a closed loop of obstruction with resultant ischemia secondary to vascular occlusion [1]. Transverse colon volvulus is an uncommon cause of intestinal obstruction. It is a surgical emergency since it can lead to bowel infarction, peritonitis, and death [2]. We report an unusual case of transverse colon volvulus presenting as intestinal obstruction and distended colon mimicking right-sided pneumothorax.

## Case presentation

### Clinical history

A 35-year-old Congolese immigrant male was admitted to Fort Portal Regional Referral Hospital with a 1-week history of colicky abdominal pain, abdominal distension, 3 days of non-bilious vomiting, and failure to pass stool and flatus. He also had difficulty in breathing that worsened over 5 days. There was no significant past medical history of previous abdominal surgeries, trauma, or chronic constipation. He did not report a similar presentation in the past. The patient had a right chest drain inserted by a junior house officer on admission after mistaking the distended right chest for a pneumothorax. The chest drain did not drain anything and there was no bubbling seen in the water seal chamber. This was later reported to the senior medical officer and a point-of-care lung ultrasound was carried out.

### General exam

The patient was alert, lying in bed with his knees flexed, having difficulty breathing, sunken eyes, dry mucous membranes, blood pressure of 130/96 mmHg, and a pulse of 97 beats/minute. There was no conjunctival pallor or jaundice. His temperature was 37.0 °C and his respiratory rate was 20 breaths/minute, with oxygen saturation (SpO2) of 86% on room air.

### Cardiorespiratory exam

The right external jugular vein was distended. The chest was hyperinflated on the right and with reduced expansion on the right. There was mild tenderness, right-sided asymmetry on respiration, left tracheal deviation, and the apex beat was in the sixth intercostal space, left anterior axillary line. There was hyperresonant percussion note over the right side of the chest with normal resonance over the left side of the chest, as well as absent breath sounds over the right side of the lung, vesicular breath sounds heard over the left side of the lung, and no added sounds. The heart sounds were normal, with no added sounds.

### Abdominal exam

The abdomen was moderately distended and not moving with respiration. There were visible bowel loops in the left upper quadrant. No organomegaly. No peristaltic movements were seen. On palpation, the abdomen was tender, and tense but without guarding or rebound tenderness. Absent bowel sounds on auscultation. A digital rectal examination revealed an empty rectum with no mass in the lumen.

## Radiology investigations

Figure 5 the X-ray on the  left (chest X-ray) shows a grossly dilated bowel loop, elevation of the right hemidiaphragm with the collapse of the right lung, and a mediastinal shift to the left. X-ray on the  right(erect abdominal X-ray) shows multiple air-fluid levels.

**Fig. 1:**
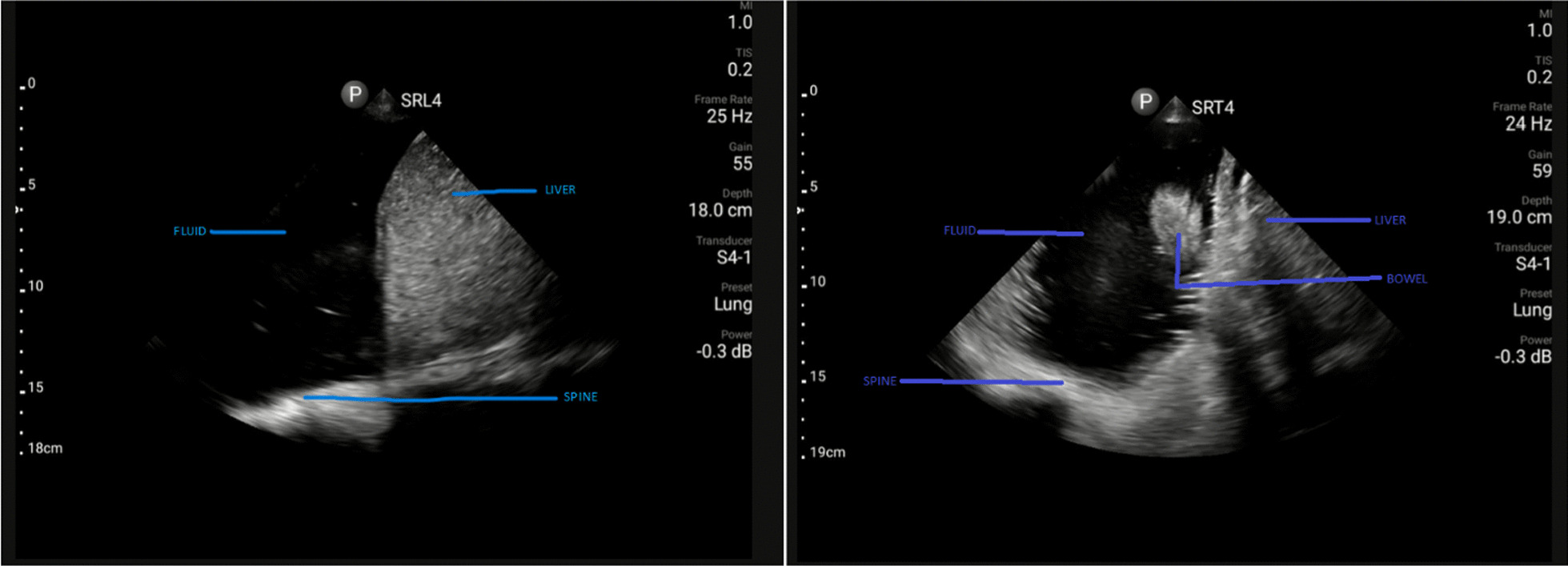
Point-of-care lung ultrasound right mid-axillary region on admission. A longitudinal view (left) and a transverse view (right) were taken

Lung point-of-care ultrasonography (POCUS) on the right upper chest showed pleural sliding and the lower right chest had dense echogenic components and fluid at the level of the diaphragm (Fig. 1). Additional files 1, 2, and 3 contain videos showing these findings on lung POCUS and SRL4 means the probe was placed longitudinally while SRT4 means that the probe was in a transverse position at the right lower axillary area.  There was pleural sliding and A lines over the left chest. The cardiac scan was normal.

The abdominal ultrasound scan done by the radiology department on admission revealed grossly distended bowel loops and no free fluid in the peritoneal cavity.

### Differential diagnosis

- Large bowel obstruction complicated by diaphragmatic hernia
- Spontaneous pneumothorax with features of tension pneumothorax

## Treatment

### Immediate

1. Fluid resuscitation by administration of normal saline and Lactated Ringer’s solution. In view of severe dehydration, an initial bolus at 20 ml/kg was administered and thereafter maintenance fluids continued using the 4:2:1 rule. Patient’s urine output of at least 1 ml/kg/hour was used to determine the adequacy of resuscitation over 4 hours.
2. Nil per oral
3. Nasogastric tube was inserted to decompress the stomach.
4. Urinary catheter was inserted to monitor urine output. An output of at least 1 ml/kg/hour was considered adequate, and in this case, 350 ml was collected in a period of 4 hours preoperatively
5. Oxygen therapy via nasal prongs at a rate of 5 l/min was administered.

With the suspicion of a diaphragmatic hernia and anticipation of the need for intensive care unit (ICU) care, the patient was then referred from Fort Portal Regional Referral Hospital to Mulago National Referral Hospital for definitive specialist surgical management.

### Definitive

Resuscitation with intravenous fluids was continued preoperatively at Mulago National Referral Hospital.

The white blood cell count was 10.00 × 10^3^/µl with normal differential count, hemoglobin 12 g/dl, platelet count (PLT) 200,000, blood grouping, and cross-matching. Serum sodium was 140 mmol/l, chloride (Cl) 100 mmol/l, and potassium (K) 3.8 mmol/l.

A decision was made to carry out an emergency exploratory laparotomy. The patient was prepared as per the hospital protocols, and then a laparotomy was carried out.

**Fig. 2:**
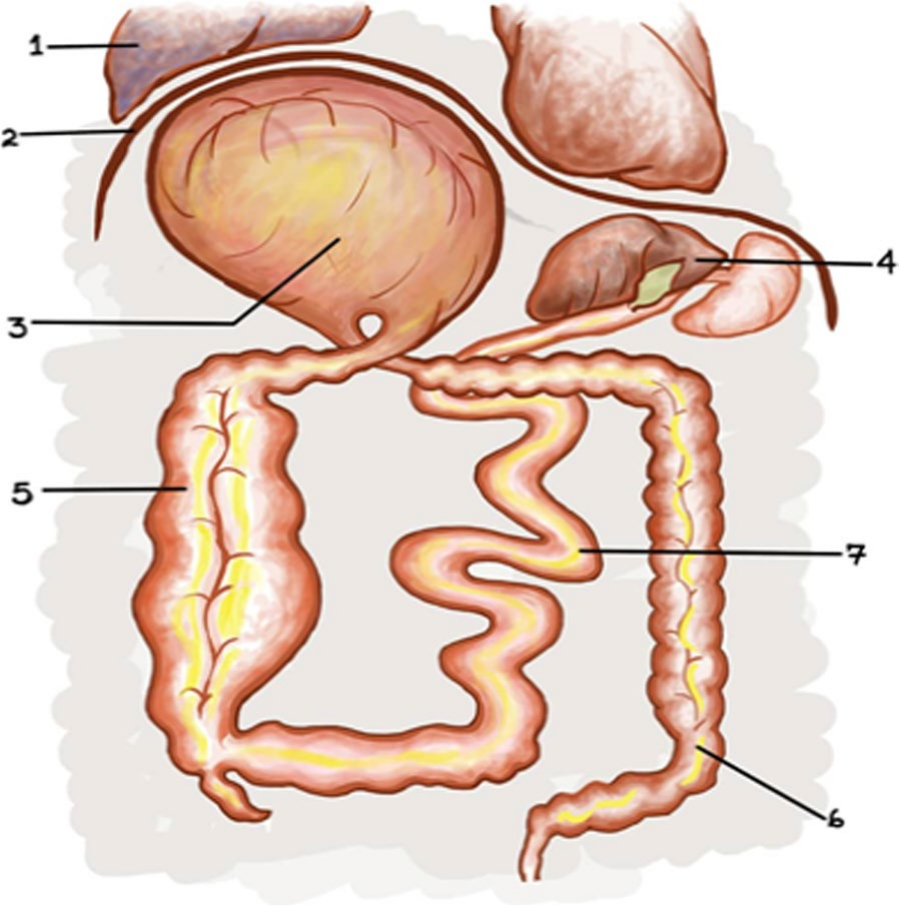
Closed loop obstruction and intraabdominal organ displacement (adapted from surgical notes). (1) Collapsed right lung, (2) upward displacement of the diaphragm, (3) transverse colon volvulus (360° counterclockwise) with massively distended closed loop, (4) left displacement of the liver and gall bladder, (5) massively distended ascending colon, (6) collapsed descending and sigmoid colon, and (7) moderately distended small bowel

Intraoperatively, the transverse colon had rotated 360 degrees counterclockwise with a massively distended and viable closed loop causing an upward displacement of the right hemidiaphragm, left displacement of the liver, and porta hepatis as shown in Fig. 2. There was also a massively distended ascending colon with several adhesions and a collapsed descending and sigmoid colon (Fig. 2). Other abdominal organs were normal.

The patient developed hemodynamic instability intraoperatively characterized by a rising pulse rate of 180–200 beats per minute, falling BP with a mean arterial pressure averaging 50–60 mmHg, and O2 saturation falling below 90%.

This necessitated damage control surgery. The transverse colon was derotated. A decompressive loop ileostomy was fashioned at about 30 cm from the ileocecal junction. The abdomen was lavaged and then closed in layers. A chest drain was inserted through an old incision in the right chest with the suspension of an iatrogenic pneumothorax. There was no obvious defect seen in the diaphragm.

The patient was then transferred to a high-dependency unit and later to the ward for continued postoperative care. Recovery was uneventful.

**Fig. 3:**
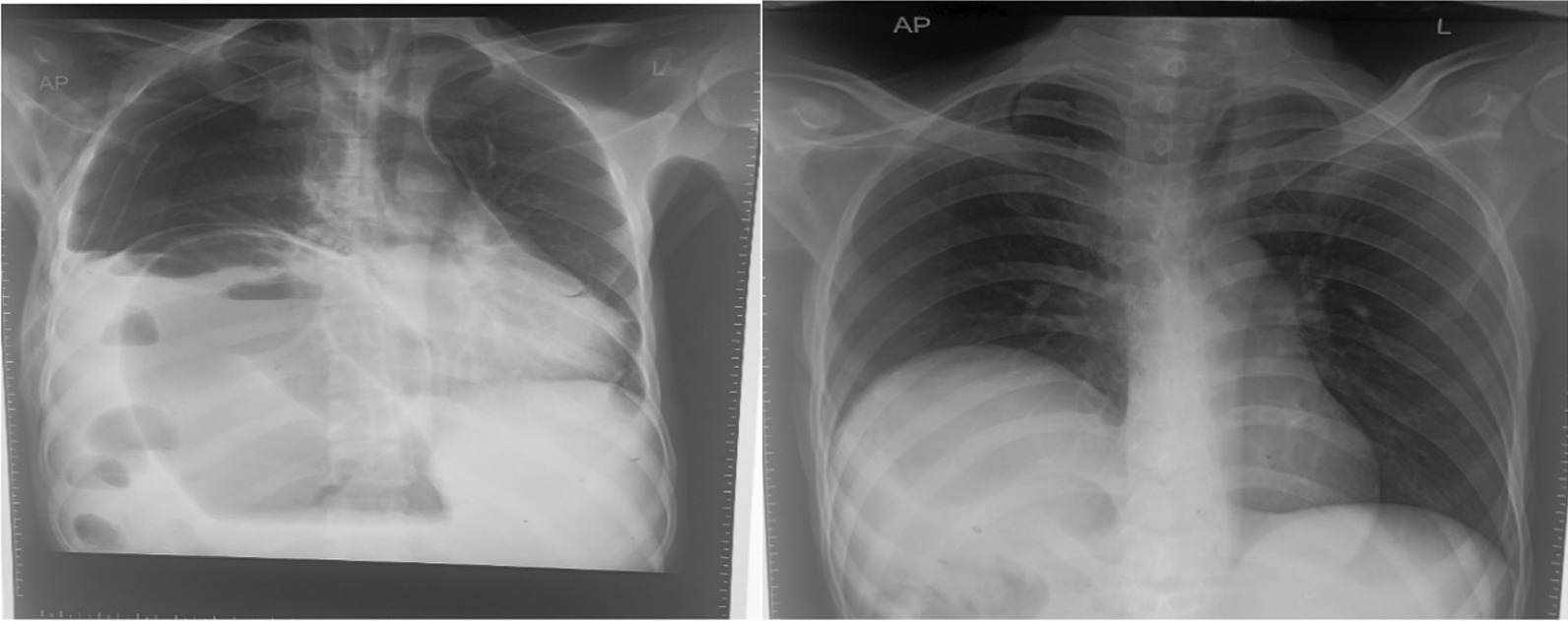
Follow-up X-rays. The image on the left shows an X-ray taken on the third postoperative day after the transverse colon was derotated and an ileostomy was placed (first operation). The image on the right shows an X-ray taken on 11 May 2022, 1 week following the transverse colon colectomy and anastomosis (second operation)

Follow-up chest X-ray (CXR) done on the third postoperative day showed no features of pneumothorax and the chest drain was later removed (Fig. 3).

The patient was discharged after 10 days and scheduled to have a second stage surgery (definitive operation) after 6 weeks.

### Follow-up (Fig. 4)

**Fig. 4:**
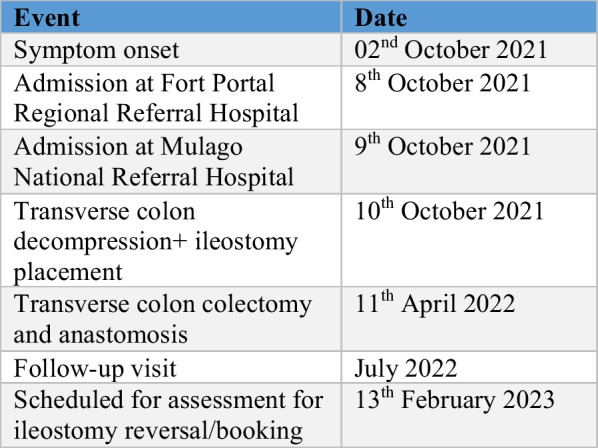
Summary of events

#### April 2022

However, he was readmitted 6 months after the first admission. On opening the abdomen, there were dense adhesions involving the small and large bowel and the stomach. The transverse colon was adherent to the right hemidiaphragm. Extensive adhesiolysis to mobilize the hepatic flexure and transverse colon from the right hemidiaphragm was done with difficulty.

The hepatic flexure and proximal two-thirds of the transverse colon were resected and an end-to-end colo-colonic anastomosis was carried out.

The ileostomy was left in place to protect the distal colonic anastomosis and it was to be reversed after 6–8 weeks.

### Follow-up: July 2022 onward

The patient was examined at the outpatient clinic and sent for nutritional assessment and rehabilitation.

On 20 September 2022, a Sudan virus disease outbreak was declared in the Mubende region, which was later put under lockdown [3]. The patient was not able to travel to Kampala and therefore the ileostomy reversal was further delayed.

He is scheduled for examination on 13 February 2023 at the surgical outpatient department.

## Discussion

### Incidence

Widespread differences based on geographic and epidemiologic factors are seen in the distribution of volvulus [4]. Intestinal volvulus is Uganda’s second most common cause of intestinal obstruction and has been on the increase during the past four decades [5]. Transverse colon volvulus incidence is comparatively rare when compared with sigmoid and cecal volvulus [4–7]. A review of 306 cases of colonic volvulus from the 1960s found that only 4% of cases involved the transverse colon [4]. To the best of our knowledge, no cases of transverse colon volvulus have been published in Uganda in the last decade.

### Etiology and mortality

A short transverse mesocolon and wide points of fixation of the hepatic and splenic flexures normally prevent the transverse colon from twisting. Congenital, mechanical, and physiologic factors can alter these relationships resulting in transverse colon volvulus [4]. Redundancy, non-fixation, or other visceral abnormalities, including Chilaiditi syndrome (hepatodiaphragmatic interposition of the colon) are the congenital properties thought to cause a volvulus [2, 4, 6, 8]. Our patient had an elongated and redundant transverse colon that was dilated, pushing over the right diaphragm. However, the colon was fixed at the hepatic and splenic flexure areas.

Physiologic factors include chronic constipation from a variety of causes, a high-fiber diet, and megacolon from Hirschsprung’s disease [4, 6, 9]. Our patient reported no history of chronic constipation.

Mechanical factors generally include some form of distal colonic obstruction, from either adhesion, neoplasm, stricture, or sigmoid volvulus [2, 4]. The patient had no history of neoplasms, adhesions secondary to previous surgeries, or sigmoid volvulus.

In patients with gangrenous bowels, the mortality rate from volvulus is higher [7]. Therefore, early diagnosis and treatment are of great value. There was no gangrene in this case; however, the patient did not present early.

## Clinical presentation

Transverse colon volvulus is categorized as either acute fulminant or subacute progressive [10]. Patients with the acute fulminant type of presentation have a sudden onset of severe abdominal pain, vomiting, minimal abdominal distension, rebound tenderness, and rapid clinical deterioration.

Subacute onset is characterized by massive abdominal distension in the setting of mild abdominal pain without rebound tenderness, nausea, or vomiting [4, 8, 9, 11]. The leukocyte count is normal or mildly elevated due to a lack of ischemia at early stages [8, 11]. Our patient presented with subacute transverse colon volvulus and also had a normal leukocyte count. This subacute presentation is what accounts for the absence of gangrene and relatively slow clinical progress.

Patients with tension pneumothorax present with dyspnea, tachypnea, and distended neck veins. The clinical exam may reveal tracheal deviation, hyper resonance, and absent breath sounds over the affected hemithorax [12]. Our patient presented with difficulty in breathing, absent breath sounds, hyperresonant percussion note over the right hemithorax, and left tracheal deviation. These findings mimic the presentation of a pneumothorax. The presence of difficulty in breathing was most likely a result of the collapsed right lung, but other factors such as acidosis and low hemoglobin count could also affect the patient’s breathing pattern. The right lung collapse was most likely caused by dilated bowel loop. Our patient had a hemoglobin count of 12 g/dl but blood gas analysis was not available at this hospital.

## Diagnosis

The diagnosis of volvulus of the transverse colon before surgery is rarely observed; it is most often made intraoperatively [6–9]. There are no characteristic radiographic features, as in the case of the volvulus of the sigmoid colon and cecum. Some authors suggested that the presence of distention of the proximal colon, with an empty distal bowel with two levels of fluid in the epigastrium in X-ray, may suggest the diagnosis [4, 8, 9].

**Fig. 5:**
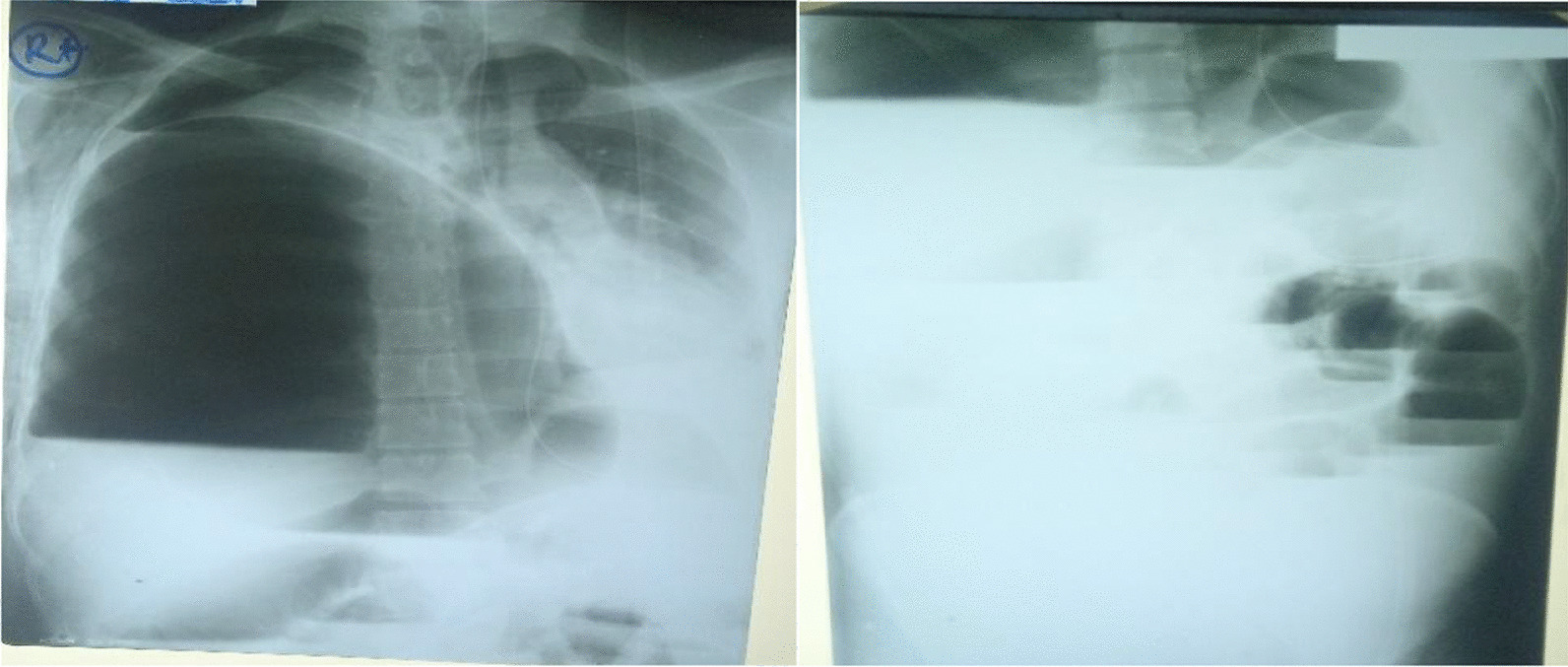
X-rays of the chest and abdomen, respectively, on admission

The patient’s chest X-ray showed severe elevation of the right hemidiaphragm secondary to the dilated loop of the colon, collapsed right lung, and shifting of the mediastinum to the left (Fig. 5). The abdominal X-ray demonstrated multiple air fluid levels (Fig. 5). We have not come across cases of transverse colon volvulus with a mediastinal shift.

Computed tomography (CT) scans are valuable tools in the evaluation of large bowel obstruction [13]. However, no CT scan image was done for our patient since there was no CT scanner at the referral site, and when he was referred to the national referral hospital and offered a CT scan, he could not afford the cost (120,000 Uganda shillings, ~ US $37). This is more than 50% of the monthly salary for a subsistence farmer such as this patient [14].

Point-of-care ultrasound (POCUS), as a non-invasive and radiation-free bedside diagnostic tool, is beneficial in critical decision-making and rapidly guiding further interventions [15]. In our case, POCUS was delayed until after the patient was admitted to the surgical ward. The lung scan on the ward demonstrated fluid on the right side, which was part of the bowel content (Fig. 1). The use of POCUS was a valuable tool in enhancing the physical exam.

### Treatment

Parallel colopexy, in which the redundant U-loop of the transverse colon is sutured to the adjacent ascending and descending limbs of the colon with a continuous absorbable seromuscular suture, has been suggested as an alternative to resection, but there are few reported cases where this procedure has been carried out [4]. Many authors recommend resection as a definitive treatment, either as a segmental colectomy or as an extended right hemicolectomy [4].

The surgical options in the management of acute large bowel obstruction, as a consequence of transverse colon volvulus, are one- or two-stage procedures. A one-stage procedure includes intraoperative colonic irrigation, resection of non-viable bowel, and primary anastomosis to avoid stoma creation.

In a two-stage procedure, two options are available: (1) bowel is resected; the proximal end is brought out as terminal colostomy and distal end as a mucus fistula; 2–3 months’ post-surgery, end-to-end anastomosis is performed and (2) bowel is resected and end-to-end anastomosis is performed; a defunctioning colostomy or a loop ileostomy is fashioned to protect the anastomosis, which is closed 3–4 weeks later.

Our patient initially underwent damage control surgery consisting of de-rotation of the viable transverse volvulus with decompressive loop ileostomy due to the hemodynamic instability intraoperatively. The surgeons chose an ileostomy over a colostomy due to wanting to limit the surgery duration for the patient.

This was followed 6 months later by segmental transverse colectomy with primary anastomosis and the initial loop ileostomy left intact as a protective/defunctioning stoma.

The 6-month delay was a result of coronavirus disease 2019 (COVID-19) restrictions on routine hospital activities. This long duration partly explains the extensive adhesions encountered during the second procedure.

Ileostomy reversal was unfortunately delayed a second time by an outbreak of Ebola disease caused by Sudan Ebolavirus in the Mubende district, and a lack of funds to come to the hospital earlier. However, the patient is scheduled for review and scheduling date for ileostomy reversal on 13 February 2023.

The two-stage procedure is preferred for bowel gangrene, perforation, and hemodynamic instability [6]. In our patient, the bowel was viable but the patient was hemodynamically unstable; thus, a two-stage procedure was carried out.

## Conclusion

Transverse colon volvulus is rare, and its presentation can mimic pneumothorax when a distended bowel loop causes upward displacement of the diaphragm and lung collapse.

The use of point-of-care ultrasonography can be helpful in ruling out pneumothorax and also in avoiding events such as unwanted chest drain insertions. COVID-19 restrictions to hospital access, the 2022 Sudan Ebola disease outbreak, and financial constraints resulted in the delay of follow-up surgeries.

## Supplementary Information

**Additional file 1.** A lung ultrasound (POCUS) video was taken when the patient was lying supine in bed, probe at the right lower axillary area in a transverse orientation. The video shows fluid (bowel contents) and dense echogenic components (bowel).

**Additional file 2.** A lung ultrasound (POCUS) video was taken when the patient was lying supine in bed, probe at the right lower axillary area in a longitudinal orientation. The video shows fluid in the right chest with a positive spine sign.

**Additional file 3.** Video 3 is the same as attachment 2 but at a deeper depth.

## Data Availability

For this case report, clinical information was obtained from patient files at Mulago National Referral Hospital and Fort Portal Regional Referral Hospital. On request, more details are available, but only in compliance with the two hospitals’ privacy rules.

## Abbreviation

POCUS: Point-of-care ultrasonography
